# Malaria endemicity and co-infection with tissue-dwelling parasites in Sub-Saharan Africa: a review

**DOI:** 10.1186/s40249-015-0070-0

**Published:** 2015-08-29

**Authors:** Nyamongo W. Onkoba, Moses J. Chimbari, Samson Mukaratirwa

**Affiliations:** College of Health Sciences, School of Nursing and Public Health, University of KwaZulu-Natal, Howard Campus, Durban, South Africa; Departmet of Tropical Infectious Diseases, Institute of Primate Research, Karen, Nairobi, Kenya; School of Life Sciences, University of KwaZulu-Natal, Westville Campus, Durban, South Africa

**Keywords:** Malaria, Tissue-dwelling helminths, Zoonosis, Co-infection, Immunomodulation

## Abstract

Mechanisms and outcomes of host-parasite interactions during malaria co-infections with gastrointestinal helminths are reasonably understood. In contrast, very little is known about such mechanisms in cases of malaria co-infections with tissue-dwelling parasites. This is lack of knowledge is exacerbated by misdiagnosis, lack of pathognomonic clinical signs and the chronic nature of tissue-dwelling helminthic infections. A good understanding of the implications of tissue-dwelling parasitic co-infections with malaria will contribute towards the improvement of the control and management of such co-infections in endemic areas. This review summarises and discusses current information available and gaps in research on malaria co-infection with gastro-intestinal helminths and tissue-dwelling parasites with emphasis on helminthic infections, in terms of the effects of migrating larval stages and intra and extracellular localisations of protozoan parasites and helminths in organs, tissues, and vascular and lymphatic circulations.

**Electronic supplementary material:**

The online version of this article (doi:10.1186/s40249-015-0070-0) contains supplementary material, which is available to authorized users.

## Multilingual abstracts

Please see Additional file 1 for translations of the abstract into the six official working languages of the United Nations.

## Introduction

Malaria is a deadly infectious disease and one of the main health problems facing developing countries in Sub-Saharan Africa (SSA) and Asia. Globally, 3.4 billion people are at risk of new malaria infections, and there are around one million deaths annually [1–3]. *Plasmodium falciparum, Plasmodium vivax, Plasmodium malariae, Plasmodium ovale* and *Plasmodium knowlesi* parasites infect humans under normal conditions [4] with *P. falciparum* and *P. vivax* being the major species that cause morbidity and mortality in children under five years of age, pregnant women and travellers from non-malarious areas [5, 6].

In SSA, morbidity and mortality due to malaria is decreasing despite a lack of a malaria vaccine, emergence of parasite resistance to available anti-malarial drugs, the anopheline mosquito being resistant to insecticide residual spraying and a poor socio-economic situation that hinders malaria control and management [7–9]. Efforts in drug discovery and vaccine development are hindered by limited knowledge of the underlying cellular and molecular mechanisms of host-parasite interactions during co-infection and polyparasitism [10, 11]. This is also aggravated by the emergence of zoonotic *P. knowlesi* malaria infections [12–14] as well as other zoonotic infectious diseases [15, 16]. Trichinellosis is an emerging and re-emerging zoonotic disease the geographical distribution of which overlaps with malaria in endemic areas of Tanzania, Uganda, Kenya, Ethiopia, Zimbabwe, South Africa and Mozambique [17–23]. The development of vaccines against parasitic infections has been complicated due to the fact that co-infecting parasites have life cycles that are either direct or complex. Direct life cycles involve cycling of mature parasites from one definitive host to another while complex life cycles involve cycling of distinct developing life stages through a number of intermediate hosts [24]. Parasite cycling within intermediate hosts causes trafficking of molecular epitopes resulting in a generation of parasites variant surface antigens and excretory and/or secretory products that act as host immuno-regulators during co-infections and clinical trials, and hinders the understanding of parasite biology [25–36].

Epidemiological studies have shown that the largest burden of malaria infections is felt by communities living in poor regions of developing countries [37–39]. In these areas, high prevalence of soil-transmitted helminthic infections have also been documented [40]. This results in co-infections, multi-parasitism or polyparasitism [41]. In the past three decades, several studies have been undertaken to establish the nature of interaction that occurs between soil-transmitted helminths (STHs) and malaria during co-infection scenarios. The studies have mainly focused on immunological aspects and disease outcomes neglecting non-immunological mechanisms that may explain the heterogeneity observed in these studies [42, 43]. Varying conclusions have been made from both epidemiological studies and laboratory experiments. Some studies have established that helminths may confer protection against cerebral malaria, others indicate that helminths exacerbate malaria, others report a reduction or increase in prevalence and transmission of malaria, while a few others report no association between the parasites [44–47]. The lack of general consensus in the studies is evidence that malaria immunity is not well understood. However, it is argued that STHs influence clinical malaria disease presentation or confer malaria tolerance through the establishment of chronic infections, induction of adaptive immunity [48] and immunosuppression of immune responses to unrelated antigens and parasites [49]. These result in an induction of host regulatory immunity, and signalling and effector mechanisms [50–52] that are beneficial to co-infecting parasites. This is mainly due to host’s failure to regulate the immune responses induced by the parasites. During co-infections, one parasite does not have direct influence on disease outcome and establishment of another parasite, however, the concept of parasite-host-parasite interactions plays a key role. One parasite influences the host to induce immune responses that will favour its establishment which in the long run, become beneficial to the co-infecting parasite. This immunological phenomenon is parasite-driven to make the host susceptible to infection and not favour the establishment of the co-infecting parasite. The amelioration or exacerbation of the disease outcome of the co-infecting parasites is a spill-over effect.

In the majority of co-infection studies, tissue-dwelling parasites, prevalent in SSA, have not been adequately considered. The hypothetical arguments presented are sketchy, making it difficult to clearly predict disease outcomes during malaria interaction with tissue-dwelling parasites. In this review, we discuss and summarise the available information and research gaps in studies undertaken on the interactions between malaria and tissue-dwelling parasites.

## Review

### Methods

#### Information sources

The online bibliographic databases, MEDLINE/PubMed, EMBASE, Web of Science, Cochrane Library and Google Scholar were searched for studies on host-parasite interactions of malaria co-infection with tissue-dwelling helminths (up to May 2015). Bibliographic lists and references of the selected papers and previous reviews were used as leads for identification of additional studies.

#### Literature search

The search was conducted using predefined medical subject heading (MeSH) terms, Boolean operators (OR, AND) and truncation symbols used in combinations of direct key words: malaria, protozoa, co-infection, nematodes, tissue-dwelling parasites, cestodes, trematodes, intracellular parasites, helminths AND all permutations of MeSH terms in all fields.

#### Study selection

Studies were included in the review if they explicitly reported on immune responses and disease outcomes during malaria co-infection with: (i) tissue and organ-dwelling parasitic protozoa; (ii) migrating parasitic helminths and protozoa; and (iii) vascular and lymphatic circulation dwelling parasites. One hundred and sixty-eight (168) studies were retrieved from the search of published work, of which 13 were excluded because of duplication and 123 were irrelevant because they were dealing with malaria co-infections and soil-transmitted parasites. Therefore, 32 studies including abstracts, reviews and reports on malaria co-infection with tissue-dwelling parasites were selected and reviewed. No grey literature was included. All articles were managed using Mendeley Desktop reference manager version 1.13.3 (NY, USA). The results of the analysis of the full papers read are described below.

## Results

### Migratory helminths and protozoans, and malaria co-infections

Poor hygiene and sanitary conditions, and dysfunctional health delivery systems in developing countries predispose people living in these areas to STH infections [53, 54]. For example, humans acquire trichinellosis through ingestion of raw or undercooked meat contaminated with infective *Trichinella* larvae [55], or gastrointestinal helminthiases/protozoa infection by ingesting food and water contaminated with embryonated eggs/cysts [19, 56, 57]. Some STHs and protozoans have complicated life cycles that involve a tissue migration phase where larvae obligately migrate through host vital organs causing tissue damage and myositis [58–61]. The tissue migrating larvae (ML) or protozoa trigger induction of immunomodulation [62] through the release of excretory and/or secretory products that act as natural stimuli for stimulation of type 2 skewed immune responses [63]. The Th2 immune responses abrogate inflammation, delay worm expulsion and initiate tissue repair [59, 64]. But knowledge of these immunological pathways and signalling are not well described indicating the need for more research to disentangle the underlying immunological scenarios that occur.

Several co-infection studies have been designed and conducted on the assumption that chronic helminths may alter malaria severity and immunity either through Th2/T regulatory lymphocyte immunomodulation, altered antibody dependent cellular inhibition, immunosuppression of pro-inflammatory activity or presence of cross reactive antibodies [47, 65]. On the contrary, Hoeve et al., (2009) [66] established that *P. chabaudi* malaria parasites are capable of altering Th2 immune responses and initiation of pulmonary tissue repair in BALB/c mice co-infected with *Nippostrongylus brasiliensis*. This indicates that the presumption that helminths always alter malaria severity and immunity is not always correct. Several laboratory experiments have also shown that malaria parasites are capable of suppressing helminth-associated immunological activation thereby exacerbating pathological outcomes caused by the ML [67]. Therefore, this shows that co-infecting helminths influence the host immunity to mediate immune responses that are beneficial to malaria parasites during co-infection. However, the underlying pathophysiological and immunological mechanisms utilised by co-infecting parasites are not completely understood despite the findings being extrapolated to explain disease outcomes in humans. Therefore, it is imperative that considerations are made on non-immunological aspects of infections such as nutrition, immunological status, vector exposure frequency and population genetics to explain the conflicting results. Furthermore, the varied immunological profiles that are elicited by ML stages in various body compartments need to be considered when explaining concepts of immunomodulation. Establishing an animal model for malaria and tissue-dwelling helminth co-infection is of utmost importance, and the use of *Trichinella* sp. as the tissue-dwelling helminth is proposed because of its adaptability to laboratory animals as well as its ease of maintenance. For example, studies could be done on the migratory pathways taken by *Trichinella* sp. and how the *Trichinella* sp. may ameliorate allergic and autoimmune diseases in mono- and co-infections with malaria [62, 68, 69]. To our knowledge there are no studies that have been undertaken to determine the interaction of *Trichinella* sp. with tropical infectious diseases. In SSA, although very few human cases have been reported, trichinellosis is considered as an emerging/re-emerging zoonotic disease that has been reported to infect a variety of mammals [19, 20, 70]. Onkoba et al. [71] established that mice co-infected with chronic *T. zimbabwensis* ameliorate and supress *P. berghei* infection. This is attributable to the comparable levels of interferon gamma (IFN-γ) secreted during co-infection and correlated with protective immunity [72]. However, further research is needed to provide new knowledge and insight into its co-infection with malaria, and the implication on vaccine efficacy and development of diagnostic tools for surveillance and control in case of future outbreaks.

### Enteric-dwelling protozoa and malaria

Intestinal protozoans, *Giardia lamblia* and *Entamoeba histolytica* have been reported to be major causes of severe intestinal disorder mostly in children, and HIV/AIDS and immunocompromised patients [73–75]. The protozoa colonise the intestinal mucosa where they elicit localised innate immune responses against severe forms of the disease [76]. The underlying mechanisms for their unusual migration in the small intestines are still unknown [77].

Coccidian parasites, *Isospora belli, Cryptosporidium* sp. and *Cyclospora* sp. also cause severe diarrhoea, morbidity and mortality [78]. Despite this, their actual disease burden and prevalence are underestimated in developing countries due to a lack of patient records and sensitive serological assays for disease detection [76]. In developing countries, prevalence of *Cryptosporidium parvum* infections are increasing due to environmental contaminations by pets, poultry, domestic animals and infected humans [79–81]. In literature, these enteric-dwelling protozoan parasites are only considered as opportunistic infections that are acquired by children, and HIV/AIDS and immunocompromised patients [82–85]. Their role as potential co-infecting parasites with tropical infectious diseases such as malaria has been neglected despite their prevalence in SSA where malaria is endemic. The risks of potential co-infection of enteric-dwelling parasites with malaria is possible through contaminated drinking and recreational water [86, 87], being in overcrowded households, coming into contact with infected calves and maintaining poor personal hygiene [82]. Enteric-dwelling parasitic infections lack specific therapy and vaccines making control of co-infections with malaria a challenge. Persons co-infected with malaria and enteric parasites are expected to exhibit severe diarrhoea, wasting syndrome and reduced quality of life, resulting in a high morbidity and mortality rate in the young and elderly, as well as immunocompromised patients. Co-infections are possible is SSA because susceptible persons live in environments contaminated with sewage, and also share housing with young calves, poultry, cats and dogs that are potential sources of zoonotic transmissions [81, 88].

### Blood and tissue-dwelling protozoan parasites

Transmissions of vector-borne parasitic infections are on the rise due to changes in climate and global trends, human behaviour, vector behaviour and prey/host switching [85, 89]. The complexity of their life cycles, sophistication in their induction of immune evasion and intricate host-parasite interactions [90] have complicated their diagnosis, drug discovery and vaccine development, as described below:

a) Trypanosomes and malaria: In SSA, the tsetse fly transmits extracellular protozoan parasites cause debilitating human African trypanosomiasis (HAT) and nagana in livestock [91]. The diseases have endemic foci in East, Southern and West Africa where they share the same geographical distributions with malaria and STHs, resulting in co-infections and polyparasitism [92]. Prevalence studies conducted in Kenya, Uganda, Tanzania and Sudan have shown that on average 70 % of HAT patients in these countries are co-infected with malaria and STHs [93–95]. This has made diagnosis and management of HAT difficult because both malaria and HAT have common clinical symptoms: intermittent fever, headache, general body pains, sleep disturbances and coma [96]. Mice concurrently infected with *P. yoelii* or *Trypanosoma brucei* have been shown to block resistance to *Echinostoma revolutum* parasite infection. [97]. This suggests that a synergistic interaction exists between protozoan and helminth infections. However, these studies do not provide explicit information on parasite-specific cellular immune and disease outcomes during these interactions, an indication that additional studies are needed. Malaria co-infection with HAT will result in exacerbation of malaria disease outcome with cerebral involvement. Both parasites potentially cross the blood brain barrier and the sequester in micro-vasculature of the brain resulting in cerebral malaria and eventually coma [91, 94, 98, 99].

b) *Babesia* sp*.* and malaria: Due to increased human-wildlife and livestock-wildlife interactions, a severe recrudescence of malaria-like babesiosis in humans and livestock has been reported [100]. In SSA, the actual prevalence and distribution of tick-borne diseases have not been well mapped [101–104]. In humans, *Babesia* sp. infections might be misdiagnosed as *Plasmodium* sp. because of their overlapping similarities in symptoms [105–109]. This setback has compromised diagnosis, treatment, management of both diseases and possible development of vaccines [78, 100, 108, 110]. During babesiosis infection, the host elicits humoral and cell-mediated immune responses that are responsible for parasite clearance. However, immune-evasion has been suspected during infection [111]. Clark and Jacobson, [112] established that both *Babesia* and *Plasmodium* parasites confer cross-protection to mice during co-infection. A human case report from Korea showed that *Babesia* parasites prolonged severity of malaria-induced haemolytic anaemia during co-infection [113]. A child from Ivory Coast co-infected with *Plasmodium* sp. and *Babesia* sp. parasites exhibited markedly enhanced malaria severity [114]. On the other hand, rhesus macaques with chronic *B. microti* infection showed that *B. microti* parasites were able to suppress *P. cynomolgi* infection [115]. Therefore, studies show that *B. microti* parasites either provoke induction of immune responses that either ameliorates or exacerbates malaria infection. However, these few available studies have not provided enough insight into immunology and cellular mechanisms that are involved during mono- and co-infection.

c) *Leishmania* sp. and malaria: In Sudan and Uganda, *Leishmania donovani* complex parasites and malaria have been reported to co-infect humans. The co-infections showed a synergistic immunological interaction characterized by enhanced Th1 immune responses [116, 117]. The *L. donovani* complex parasites naturally colonise macrophages to initiate counter regulation of host immune responses resulting in a release of anergic/dysfunctional T-cells and blocking of intracellular cytokine signalling in macrophages and dendritic cells [116]. Currently, the available information on the interaction of visceral leishmaniasis and malaria co-infections among pastoral communities of Kenya, Uganda and Sudan is limited. It does not provide vital information on disease outcomes and immunological interactions. Malaria-infected red blood cells are recognised and internalised macrophages and dendritic cells that are also colonised by *Leishmania* parasites. This shows that during co-infection the control of malaria will be impaired in that the effector cells are used by the *Leishmania* parasites for immunoregulation. This will result in the exacerbation of malaria disease and suppression of *Leishmania* parasites or, conversely, the parasites will impact the host immunity and influence infection and pathophysiological responses of both parasites. The role of *Leishmania* parasite mediators and vector saliva components in mediating immunosuppression of host regulatory immune responses are still unknown [118, 119]. Therefore, further research should be undertaken to determine disease prevalence and impact on socio-economic and environmental factors in regions where congruency of the two parasites is eminent.

d) *Toxoplasma gondii* and malaria: *Toxoplasma gondii* is a cosmopolitan intracellular apicomplexan parasite that causes ocular, congenital, neurological and systemic infections in approximately one third of the world’s population [120–122]. Humans acquire infection through ingestion of sporulated oocysts and trophozoites in undercooked meat, organ transplants from infected donors or through vertical transmission during pregnancy [123]. Stray dogs and cats feeding on offal at abattoirs, poor sewerage systems and sanitation standards, and anthropogenic, climatic and socio-cultural factors have been implicated in human outbreaks of *T. gondii* infections [122, 124, 125]. The severity of infection depends on host immunity and inflammatory foci involved [126]. A questionnaire study conducted in Kenya established that sources of drinking water and disposal of cat faeces are infection risks amongst subsistence farmers [127]. However, the protozoan parasite is still regarded as an opportunistic agent and not as a causative agent of major infections [84, 128–130]. This implies that toxoplasmosis and malaria co-infection cannot be ruled out in this malaria-endemic region. In several mono-infection studies, it has been established that *T. gondii* and *Plasmodium* parasites utilise similar cellular mechanisms and biochemical pathways for their nutrition, metabolism, pathology and immunomodulation [131, 132]⁠. This might indicate that during co-infections the parasites will result in competitive establishment that may promote or hamper parasite pathogenicity, and foetal and birth outcomes during pregnancy [133]⁠, severity of anaemia and mortality [124, 134–136], and severity of neurological and cerebral involvement [137, 138, 98]. Malaria and *Toxoplasma* parasites sequester in the placenta resulting in placental disc plate damage thus influencing foetal and pregnancy outcomes [139]. Despite these prospects of fatal disease outcomes there is striking paucity of information on immunological and disease outcomes and interactions during co-infections with *T. gondii*.

### Lymphatic-dwelling filarial worms and malaria

Lymphatic filariasis (LF) caused by *Wuchereria bancrofti*, *Brugia malayi*, *Onchocerca volvulus* and *Loa loa* are endemic in SSA [140, 141]. The filarial nematode worms and *Plasmodium* parasites are transmitted by the same anopheline mosquito vector making co-endemicity a common phenomenon [142, 143] This necessitates implementation of integrated control measures [141]. Several studies on chronic LF interactions with malaria have been conducted [140] and have shown that patent filariasis is able to modify immunological balance to confer protection against malaria severity or exacerbate it [144–146]. The amelioration malaria severity is achieved by the combined induction of Th1 and Th2 immune responses with increased interleukin (IL)-5 and IFN-γ production [48, 147, 148]. On the other hand, pre-patent filariasis exacerbates malaria severity through immunosuppression of IFN-γ and initiation of activation of CD4 + CD25 + FoxP3+ T-regulatory cells [145]. In epidemiological studies, antihelminthic treatment against LF has been shown to reduce LF exposure through interruption of its transmission dynamics [149, 150]. However, in murine studies, it has been shown to exacerbate malaria and sepsis [151], thus negating its usefulness in malaria-endemic areas. On the other hand, Aliota et al. [152] established that filarial worms are capable of reducing *Plasmodium* parasite infectivity within the mosquito vector. However, these studies have not determined the immunological changes that occur during deworming and its benefits towards integrated control strategies in malaria-endemic areas.

### *Taenia solium, Echinococcus granulosus and E. multilocularis,* and malaria

Larval stages of some tapeworms cause fatal liver, brain and lung metastasis in humans and livestock [153, 154]. In SSA, exposure risks are attributed to changes in human culinary habits [85, 155] and environmental contamination by stray dogs and cats [127, 156–158]. The resultant diseases cause physical damage to vital body organs and tissues, and even lead to neurological and cerebral damage [159]. Active infection favours induction of a Th2 skewed immune response characterised by markedly elevated levels of IL-4 and IL-10 [160, 161]. In several areas of SSA, tapeworm infections are rare due to religious and agricultural practices [162]. However, isolated cases of human infections have been reported in people working in commercial pig farms [163, 164], or living in areas where there are no sanitation facilities and the presence of free roaming pigs [165, 166]. Information on malaria and cestodes infections is non-existent. Therefore, further studies are needed to determine the actual prevalence, disease burdens and even cases of co-infection with malaria.

### Trematodes and malaria

*Fasciola hepatica* and *F. gigantica* in humans are emerging infections and occur in malaria endemic areas despite the parasites not being considered to have relevance in malaria co-infections [167–169]. One of the most important and common snail-borne trematode infections in humans is due to *Schistosoma haematobium* and *S. mansoni*, and these are becoming emerging or re-emerging infections in developing countries of SSA due to climate changes that are influencing spatial distribution of fresh water snails [170, 171]. Therefore, the impact of fascioliasis and schistosomiasis on communities demand rapid action and research to define control measures, transmission patterns and epidemiological situations. There is paucity of data on the interaction of fascioliasis with malaria except for shared zonal distributions in Egypt [172]. Thus, research on the interactions between both parasites in their shared eco-epidemiological settings is required.

Several laboratory experiments and epidemiological studies have been conducted and meta-analysis reviews have been done to determine the host-parasite interactions of malaria and schistosomes during co-infection [173]. The studies have shown that clinical outcomes of malaria and immune responses during co-infections with schistosomes are influenced by age, host genetics, immunity and exposure rates in humans [174–181]. In animal models, immunological responses induced also depend on the strain of the parasite and patency of the helminthic infections [182–184]. Research findings show that schistosomes induce Th2 immune responses that are either detrimental or beneficial to the host during co-infection [177, 185, 186]. These findings further show that schistosomes increase malaria susceptibility and transmission [187] but have not been able to conclusively explain the underlying mechanism and pathways for immunomodulation.

## Conclusion

There are few studies that are directed towards elucidating the host-parasite interactions and disease outcomes that are elicited by tissue-dwelling parasites during co-infection with malaria. This has created a glaring paucity of data on understanding the mechanisms and outcomes of tissue-dwelling parasites and mono- and co-infection with malaria. This has also hampered diagnosis, vaccine development, drug discovery, and management and control of these emerging and re-emerging parasites. Therefore, further studies are imperative to address this lack of data and the heterogeneity of results reported during STHs, schistosomes and filarial worm co-infection with malaria. These future studies should be designed and controlled towards elucidating cellular and molecular pathways as well as migratory pathways that are utilised by migrating tissue-dwelling helminths and protozoa. The utilisation of different study designs and approaches, as well as different tissue-dwelling helminths and protozoa will provide vital information that can be extrapolated to humans. These studies and experiments will also provide information on non-immunological aspects, timing and order of parasite infections. The disease outcomes across broad range of hosts and parasites will show evidence of parasite-host-parasite interactions at the phenotypic level. This data will be useful in explaining the actual cellular and molecular mechanisms and signalling pathways that influence conferring of protective immunity, exacerbation and/or amelioration of disease outcomes that have been observed in concomitant and concurrent infections. In the long term, the studies will provide the thrust for deworming, surveillance, diagnosis, vaccination campaigns and vaccine trials in areas of SSA where tissue-dwelling parasites are co-endemic with malaria.

## Additional file

Additional file 1:
**Multilingual abstracts in the six official working languages of the United Nations.** (PDF 255 kb)

## Abbreviations

HAT: human African trypanosomiasis
HIV/AIDS: human immunodeficiency virus infection/acquired immune deficiency syndrome
IFN-γ: interferon gamma
IL: interleukin
LF: lymphatic filariasis
MeSH: medical subject headings
ML: migrating larvae
SSA: Sub-Saharan Africa
