# Supplementary material for: Malaria endemicity and co-infection with tissue-dwelling parasites in Sub-Saharan Africa: a review
Source: Infect Dis Poverty. 2015 Aug 29;4:35. doi: 10.1186/s40249-015-0070-0 (PMC4571070; doi:10.1186/s40249-015-0070-0)
Supplement: Additional file 1: — Multilingual abstracts in the six official working languages of the United Nations. (PDF 255 kb) [file 40249_2015_70_MOESM1_ESM.pdf]

Translation of the abstract into the six official working languages of the United Nations

توطنية الملاريا والعدوى المرافقة بالطفيليات التى تقطن الأنسجة في جنوب الصحراء الكبرى الأفريقية: مراجعة

أونكوبا و. نيامونجو، موسى جـ. شمبارى و سامسون موكاراتيرواء

الملخص

إن آليات ونتائج التفاعلات بين العائل والطفيل فى حالات العدوى المرافقة بالملاريا والديدان الطفيلية المعوية تعتبر مفهومة بشكل معقول ولكننا في المقابل نعرف القليل جدا عن مثل هذه الآليات في حالات العدوى بالملاريا والطفيليات التى تقطن الأنسجة، حيث يؤدى التشخيص الخاطئ وعدم وجود علامات إكلينيكية واصمة والطبيعة المزمنة لعدوى الديدان الطفيلية التي تقطن الأنسجة إلى تفاقم هذا النقص في المعرفة، والفهم الجيد لآثار العدوى الطفيلية لأنسجة المصابين بالملاريا قد يساهم في تحسين مكافحة وإدارة مثل هذا النوع من العدوى المرافقة في الأماكن الموبوءة بالملاريا، وقد لخص الاستعراض الحالى وناقش المعلومات المتاحة والثغرات الموجودة في البحوث المتعلقة بالعدوى المرافقة بالملاريا والديدان المعوية أوالطفيليات التي تقطن الأنسجة مع التركيز على عدوى الديدان الطفيلية من حيث الآثار المترتبة على تنقل مراحل الطفيل اليرقية داخل جسم العائل، وتواجد الطفيليات وحيدة الخلية والديدان الطفيلية داخل وخارج الخلايا في الأعضاء والأنسجة ومجارى الأوعية الدموية واللمفاوية.

Translated from English version into Arabic by Mohamed R. Habib, through

CHINESE CENTER FOR DISEASE CONTROL AND PREVENTION
NATIONAL INSTITUTE OF PARASITIC DISEASES

撒哈拉以南非洲地区疟疾地方性流行情况及其与组织内寄生虫的合并感染

Onkoba W. Nyamongo, Moses J. Chimbari, Samson Mukaratirwa

摘要

在疟疾与胃肠道寄生虫合并感染的过程中，宿主-寄生虫相互作用的机制及其结果都得到合理的阐释。相比之下，疟疾与组织内寄生虫合并感染的机制却知之甚少。误诊、临床病症不明显和组织内蠕虫自然慢性感染加剧了这类知识的缺乏。对组织内寄生虫合并感染疟疾的深入理解将有助于提高流行地区该类合并感染的防控和管理水平。本文以蠕虫感染为重点，从迁移幼虫期的影响，寄生原虫和蠕虫在器官、组织、血管及淋巴循环的细胞内、外定位等方面总结和讨论了疟疾合并感染胃肠道蠕虫和组织内寄生虫的研究进展和研究差距。

Translated from English version into Chinese by Chen Jin, edited by Yang Pin, through

CHINESE CENTER FOR DISEASE CONTROL AND PREVENTION
NATIONAL INSTITUTE OF PARASITIC DISEASES

Paludisme endémique et co-infection par des parasites tissulaires en Afrique subsaharienne : revue

Onkoba W. Nyamongo, Moses J. Chimbari, Samson Mukaratirwa

RÉSUMÉ

Les mécanismes des interactions hôte-parasite au cours des co-infections entre paludisme et helminthiases gastro-intestinales et leur issue sont relativement bien compris. En revanche, on sait très peu de chose sur les mécanismes en cause dans les co-infections par le paludisme et les parasites infestant les tissus. Ce manque de connaissances est aggravé par les erreurs de diagnostic, l'absence de signes cliniques pathognomoniques et le caractère chronique des helminthiases tissulaires. Or une bonne compréhension des implications de ces co-infections contribuerait à une meilleure lutte et une meilleure gestion de ces affections dans les zones d'endémie. Notre revue résume et discute les informations actuellement disponibles et les lacunes de la recherche sur les co-infections par le paludisme et les helminthiases gastro-intestinales et tissulaires. Elle met l'accent sur les helminthiases en termes d'effets des migrations des stades larvaires et des localisations intra- et extracellulaires des protozoaires parasites et des helminthes dans les organes, les tissus et la circulation sanguine et lymphatique.

Translated from English version into French by Suzanne Assenat, through

Эндемичность малярии и коинфекция тканевыми паразитами в странах Африки южнее Сахары: Обзор

Онкоба Б. Ньямонго, Мозес Дж. Чимбари, Самсон Мукаратирва

АВТОРЕФЕРАТ

Механизмы и результаты взаимодействия хозяин-паразит при сопутствующем малярии инфицировании желудочно-кишечного тракта гельминтами достаточно хорошо изучены. В отличие от этого, очень мало известно о таких механизмах в случаях сопутствующего малярии инфицирования тканевыми паразитами. Этот недостаток знаний усугубляется неправильной диагностикой, отсутствием патогномоничных клинических признаков и хроническим характером инфекций тканевыми гельминтами. Хорошее понимание последствий тканевых паразитарных инфекций, сопутствующих малярии, будет способствовать улучшению контроля и управления такими коинфекциями в эндемичных районах. В данном обзоре обобщается и обсуждается имеющаяся информация и пробелы в исследованиях малярии, сопровождаемой инфекциями желудочно-кишечного тракта гельминтами и тканевыми паразитами с акцентом на гельминтозы с точки зрения последствий миграции личинок и внутри- и внеклеточных этапов локализации простейших паразитов и гельминтов в органах, тканях и системах обращения крови и лимфы.

Translated from English version into Russian by Yuri Geifman, through

Endemismo de la malaria y co-infección con parásitos de los tejidos en Africa Subsahariana: una revisión

Onkoba W. Nyamongo, Moses J. Chimbari, Samson Mukaratirwa

RESUMEN

Los mecanismos y resultados de las interacciones anfitrión-parásito en las co-infecciones de malaria con helmintos gastrointestinales se entiende de manera razonable. Por el contrario, poco se sabe sobre ciertos mecanismos en casos de co-infecciones de malaria con parásitos de los tejidos. Esta falta de conocimiento se ve exacerbada por diagnósticos erróneos, falta de signos clínicos patognomónicos y la naturaleza crónica de las infecciones por helmintos en los tejidos. Un buen entendimiento de las implicaciones de las co-infecciones por parásitos de los tejidos con malaria contribuirán a mejorar el control y manejo de dichas co-infecciones en zonas endémicas. Esta revisión resume y analiza la información actual disponible y las brechas en la investigación sobre la co-infección de malaria y helmintos gastrointestinales y parásitos de los tejidos con énfasis en las infecciones por helmintos, en términos de los efectos de las etapas de las larvas migratorias y las localizaciones intra y extracelulares de parásitos protozoarios y helmintos en órganos, tejidos y la circulación vascular y linfática.

Translated from English version into Spanish by Maria Alejandra Aguada, through

TRANSLATORS
WITHOUT BORDERS
